# Enzalutamide inhibits testosterone-induced growth of human prostate cancer xenografts in zebrafish and can induce bradycardia

**DOI:** 10.1038/s41598-017-14413-w

**Published:** 2017-10-31

**Authors:** Nicole Melong, Shelby Steele, Morgan MacDonald, Alice Holly, Colin C. Collins, Amina Zoubeidi, Jason N. Berman, Graham Dellaire

**Affiliations:** 1 Department of Pediatrics, IWK Health Centre, Halifax, Canada; 2 Department of Pathology, Dalhousie University, Halifax, Canada; 3 Department of Pediatrics, Dalhousie University, Halifax, Canada; 4 Department of Urology, Dalhousie University, Halifax, Canada; 5 Vancouver Prostate Centre, Vancouver, Canada

## Abstract

The zebrafish has become a popular human tumour xenograft model, particularly for solid tumours including prostate cancer (PCa). To date PCa xenotransplantation studies in zebrafish have not been performed in the presence of testosterone, even when employing androgen-dependent cell models, such as the LNCaP cell line. Thus, with the goal of more faithfully modelling the hormonal milieu in which PCa develops in humans, we sought to determine the effects of exogenous testosterone on the growth of LNCaP, or androgen-independent C4-2 cells xenografted into zebrafish embryos. Testosterone significantly increased engrafted LNCaP proliferation compared to control xenografts, which could be inhibited by co-administration of the anti-androgen receptor drug, enzalutamide. By contrast, C4-2 cell growth was not affected by either testosterone or enzalutamide. Enzalutamide also induced bradycardia and death in zebrafish embryos in a dose-dependent manner and strongly synergized with the potassium-channel blocking agent, terfenadine, known to induce long QT syndrome and cardiac arrhythmia. Together, these data not only indicate that testosterone administration should be considered in all PCa xenograft studies in zebrafish but also highlights the unique opportunity of this preclinical platform to simultaneously evaluate efficacy and toxicity of novel therapies and/or protective agents towards developing safer and more effective PCa treatments.

## Introduction

Prostate cancer (PCa) continues to be the most common cancer in Canadian men and the third leading cause of cancer death among Canadian men (Canadian Cancer Society, 2016). With advanced prostate cancer carrying such a poor prognosis, advancements of medical therapies have been highly sought after. Due to the dependence of prostate cancer growth on androgens, therapy for aggressive prostate cancer involves androgen deprivation therapy (ADT) by surgical or medical castration^1^.

Medical (i.e. chemically induced) ADT is the most commonly employed method of castration, primarily due to patient preference^2^. The two primary medical methods used are luteinizing hormone-releasing hormone (LHRH) agonists (eg. leuprolide and goserelin) and LHRH antagonists (eg. degarelix) (NIH, National Cancer Institute, 2014). LHRH agonists are synthetic proteins that are structurally similar to LHRH and bind to the LHRH receptor in the pituitary, which in turn will cause androgen production by the testes. Ultimately, this increase in androgen production will cause the pituitary to stop the production of luteinizing hormone (LH) and eventually lower testosterone to a level similar to a surgically castrated patient. When an LHRH agonist is used, there is an initial rise of serum testosterone due to a transient rise in LH, which can cause a period of testosterone-driven cancer proliferation^3^. In this case, androgen antagonists such as enzalutamide can be used for short-term blockade of this proliferation until serum LH levels fall^4,5^. Enzalutamide (also known as MDV3100) acts as an anti-androgen by directly binding to the androgen receptor (AR), preventing its nuclear translocation as well as co-activator recruitment in the ligand-receptor complex^6^. Although exhibiting potent inhibition of androgen-dependent PCa cell growth, enzalutamide is generally employed as a treatment for castration-resistant PCa (CRPC) that does not respond to ADT.

With the development of numerous treatment options for targeting the androgen receptors^7^, an efficient *in vivo* method for evaluating the efficacy of new drug therapies is highly valuable. Human tumour xenotransplantation (XT) in zebrafish has been shown to offer a unique, rapid and high throughput ability to monitor *in vivo* drug-tumour interactions^8–11^. Both the zebrafish and xenotransplanted human cells are responsive to compounds dissolved in their aquatic medium, and the transparent nature of zebrafish embryos enables the rapid visualization of tumour migration and proliferation *in vivo*. To date there have been only a few studies involving XT of PCa in zebrafish^12–14^, and despite PCa being a hormone-driven cancer, these studies did not address the impact of androgens on PCa cell growth in engrafted fish. Therefore, to directly test the impact of androgens on PCa xenografts in zebrafish, we injected the androgen-dependent LNCaP and C4-2 androgen-independent human PCa cell lines into zebrafish embryos and treated injected fish with exogenous testosterone, with and without enzalutamide. While testosterone significantly promoted the proliferation of LNCaP cells, enzalutamide was effective at restricting LNCaP proliferation both in the presence and absence of testosterone. In contrast, the proliferation of C4-2 cells, which are a model of CRPC, was not affected by treatment of testosterone or enzalutamide. Increasing concentrations of enzalutamide, however, caused significant bradycardia in these fish, which was exacerbated in the presence of terfenadine, a compound well known for inducing long QT syndrome in human patients. This study is the first to demonstrate the importance of androgen supplementation in the context of studying prostate cancer in zebrafish, positioning this model system for the evaluation of anti-androgen therapies, as well as therapy-associated cardiac dysfunction from these agents.

## Results

### Testosterone can increase the proliferation of LNCaP cells in an AR-dependent manner but not C4-2 cells engrafted in zebrafish embryos

**Figure 1:**
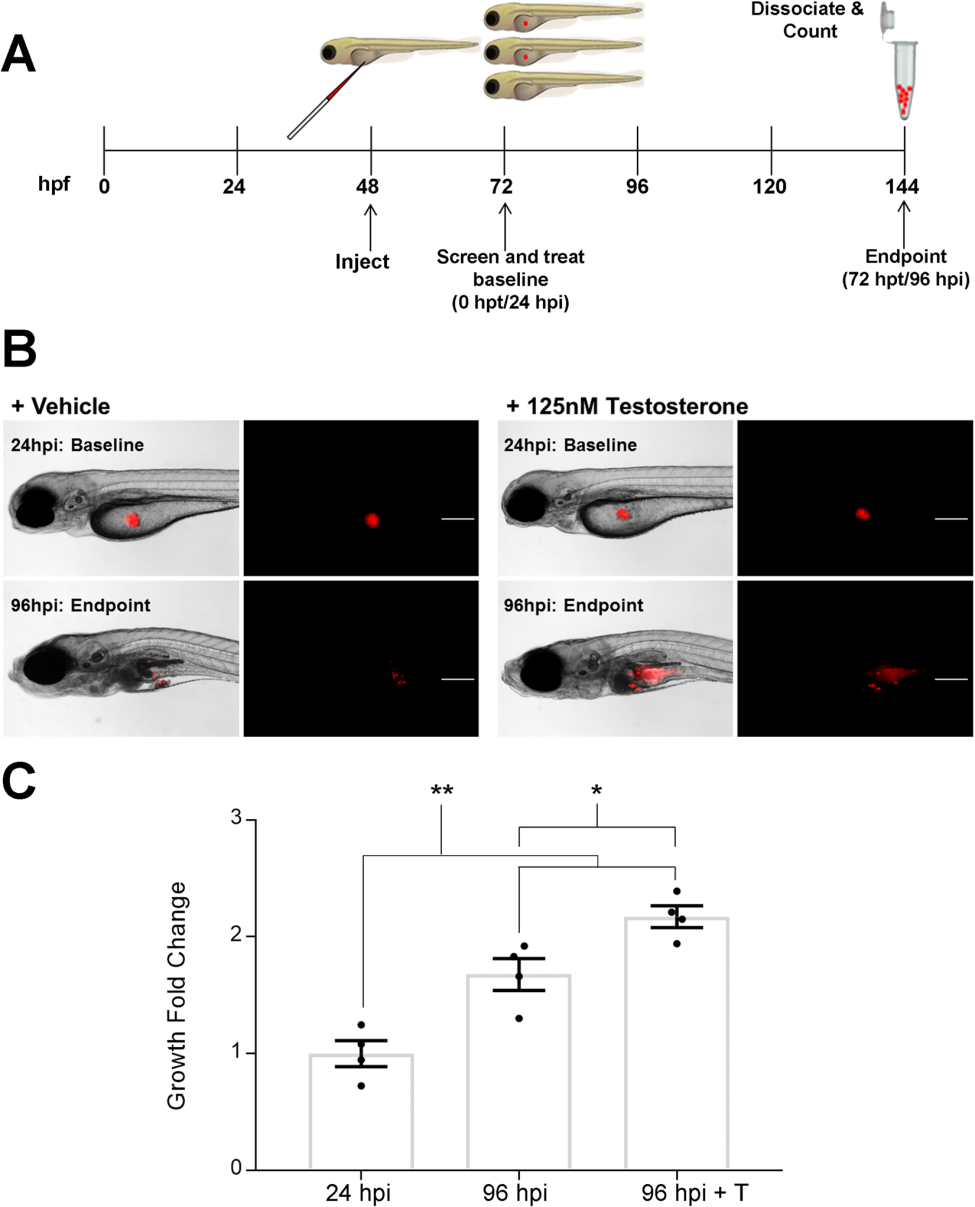
Xenografted LNCaP cells proliferate significantly *in vivo* with the addition of testosterone. (**A**) Schematic of *in vivo* zebrafish XT microinjection and cell proliferation assay. (**B**) Representative brightfield and fluorescent images of *casper* embryos injected with CMTMR labeled LNCaP cells at 24 hpi (baseline) and 96 hpi (endpoint) without and with the addition of testosterone. Scale bar = 200 microns. (**C**) Quantification of XT LNCaP cell engraftment and fold change *ex vivo* without and with the addition of 125 nM testosterone (T). LNCaP cells engrafted and proliferated in the XT model indicated by the significant increase in the fold change from baseline numbers (24 hpi) to the endpoint (96 hpi), which increased even more with the addition of testosterone. Error Bars = Mean ± SEM (N = 4); *P < 0.05, **P < 0.01 for significant increase in number of cells determined using the Student’s t-test. Groups of 20 embryos were sacrificed per replicate.

We have previously shown that a zebrafish human cancer XT platform can robustly detect and quantify the *in vivo* inhibition of leukemia cell proliferation using targeted therapies^9,10,15^. This approach has several advantages provided by the zebrafish model, including the conserved genetics and imaging opportunities inherent in the zebrafish embryo to enable studies of human PCa in an *in vivo* model that is more cost effective and complementary to murine models. Previous PCa XT studies in zebrafish did not examine the effects of exogenous androgens^12–14^. Therefore, we sought to determine the *in vivo* growth characteristics of the androgen-dependent LNCaP PCa cell line after XT in zebrafish embryos in the presence or absence of testosterone. To this end, we first determined the maximum tolerated dose (MTD) of testosterone on uninjected 72 hour post-fertilization (hpf) *casper* embryos. Embryos were treated with increasing concentrations of testosterone for a total of 72 hours. Using this approach, we determined that the MTD was ~250 nM testosterone (i.e. the first dose for which we see 80% survival), and thus for all further experiments we employed 125 nM testosterone or 50% of the MTD (i.e. MTD50^10^). For xenograft studies, groups of twenty 48 hpf embryos were injected with CellTracker orange CMTMR labelled LNCaP cells that were later sacrificed for *ex vivo* enumeration of fluorescent PCa cells at 24 hours post-injection (hpi) (i.e. 0 hours post-treatment (hpt)) and at 96 hpi (i.e. 72 hpt) (see Fig. 1A for the experimental design)^10^. We found that engrafted LNCaP cells proliferated significantly more with the addition of 125 nM testosterone (2.2 ± 0.1 fold increase between 0 and 72 hpt) compared to the vehicle control (1% DMSO) treated group (1.7 ± 0.2 fold increase) (Fig. 1B,C; p < 0.02).

**Figure 2:**
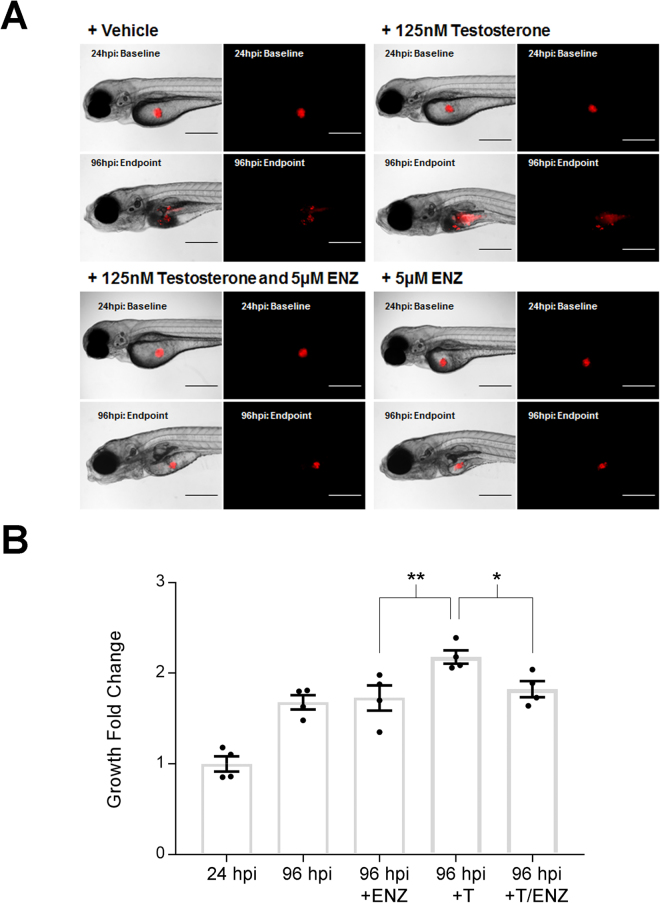
Enzalutamide inhibits testosterone-induced proliferation of xenografted LNCaP cells *in vivo*. (**A**) Representative brightfield and fluorescent images of *casper* embryos injected with CMTMR labeled LNCaP cells at 24 hpi (baseline) and 96 hpi (endpoint) without and with the addition of enzalutamide (5 µM) alone or with testosterone (125 nM). Scale bar = 200 microns. (**B**) Quantification of fluorescently labeled XT LNCaP cells showed that the addition of 5 µM of enzalutamide (ENZ) significantly decreased cell proliferation caused by the addition of 125 nM of testosterone (T) in the zebrafish model. Error Bars = Mean ± SEM (N = 4); *P < 0.05, **P < 0.01 for significant differences in cell numbers determined using the Student’s t-test. Groups of 20 embryos were sacrificed per replicate.

Since LNCaP cells express the androgen receptor, we next sought to determine if AR-dependent signaling was responsible for the proliferative effects of testosterone on these cells post-XT in the zebrafish by supplementing the embryo water with the AR-antagonist, enzalutamide. LNCaP XT embryos were treated with vehicle (1% DMSO), 5 µM enzalutamide (the zebrafish embryo MTD50), 125 nM testosterone or a combination of both (Fig. 2). Enzalutamide alone did not inhibit LNCaP growth after zebrafish XT (1.7 ± 0.3 fold increase between 0 and 72 hpt) when compared to vehicle treated embryos (1.7 ± 0.2 fold increase) and testosterone treatment again significantly increased proliferation as compared to vehicle (2.2 ± 0.2 fold increase). However, we found that co-treatment of engrafted embryos with enzalutamide could significantly block the proliferative effects of 125 nM testosterone (1.8 ± 0.2 fold increase; p < 0.03) (Fig. 2). Together, these data indicate that administration of testosterone to the embryo water does significantly increase the proliferation of LNCaP cells in engrafted zebrafish and that this proliferation is dependent on AR-signalling.

**Figure 3:**
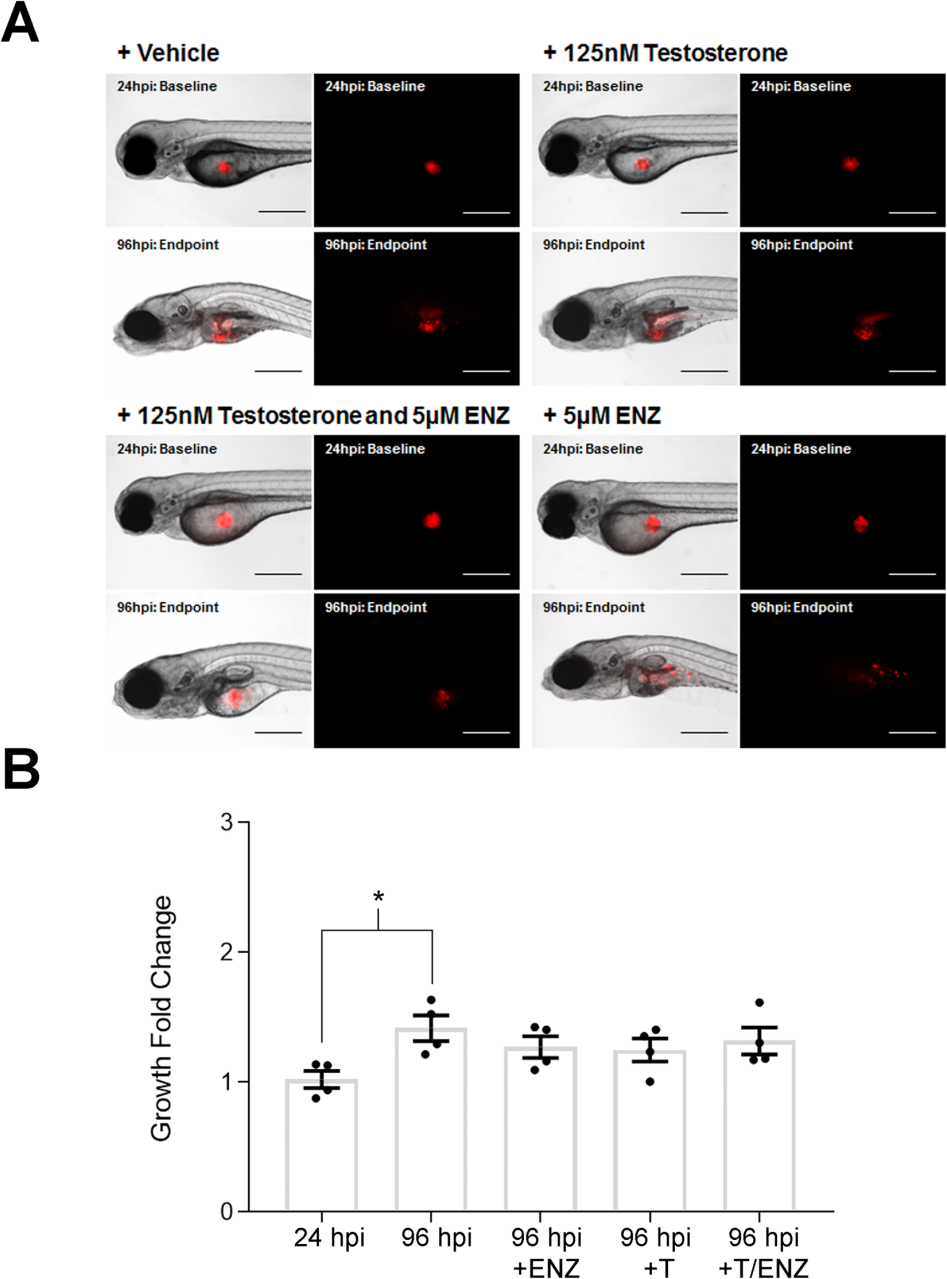
The proliferation of xenografted C4-2 CRPC cells *in vivo* is not affected by testosterone or enzalutamide. (**A**) Representative brightfield and fluorescent images of *casper* embryos injected with CMTMR labeled C4-2 cells at 24 hpi (baseline) and 96 hpi (endpoint) without and with the addition of testosterone (125 nM), enzalutamide (5 µM) or combined treatment. Scale bar = 200 microns. (**B**) Quantification of fluorescently labeled XT C4-2 cells showed that the addition of 5 µM of enzalutamide (ENZ), 125 nM of testosterone (T) or a combination of both compounds had no significant effect on cell growth in the zebrafish model. Error Bars = Mean ± SEM (N = 4); *P < 0.05 for significant differences in cell numbers determined using the Student’s t-test. Groups of 20 embryos were sacrificed per replicate.

We also examined the effects of testosterone on the growth of a xenografted CRPC cell line, C4-2, in zebrafish (Fig. 3). C4-2 cells are an androgen-independent derivative of LNCaP isolated from castrated mice engrafted with serially transplanted LNCaP cells^16^ that have low AR expression and are not growth inhibited *in vitro* by enzalutamide^17^. Although C4-2 cells did demonstrate significant cell growth *in vivo* after engraftment in zebrafish, cell growth was not enhanced by testosterone treatment, nor was growth inhibited by enzalutamide treatment in the presence or absence of testosterone (Fig. 3).

### Enzalutamide induces bradycardia and embryo death in a dose-dependent manner

**Figure 4:**
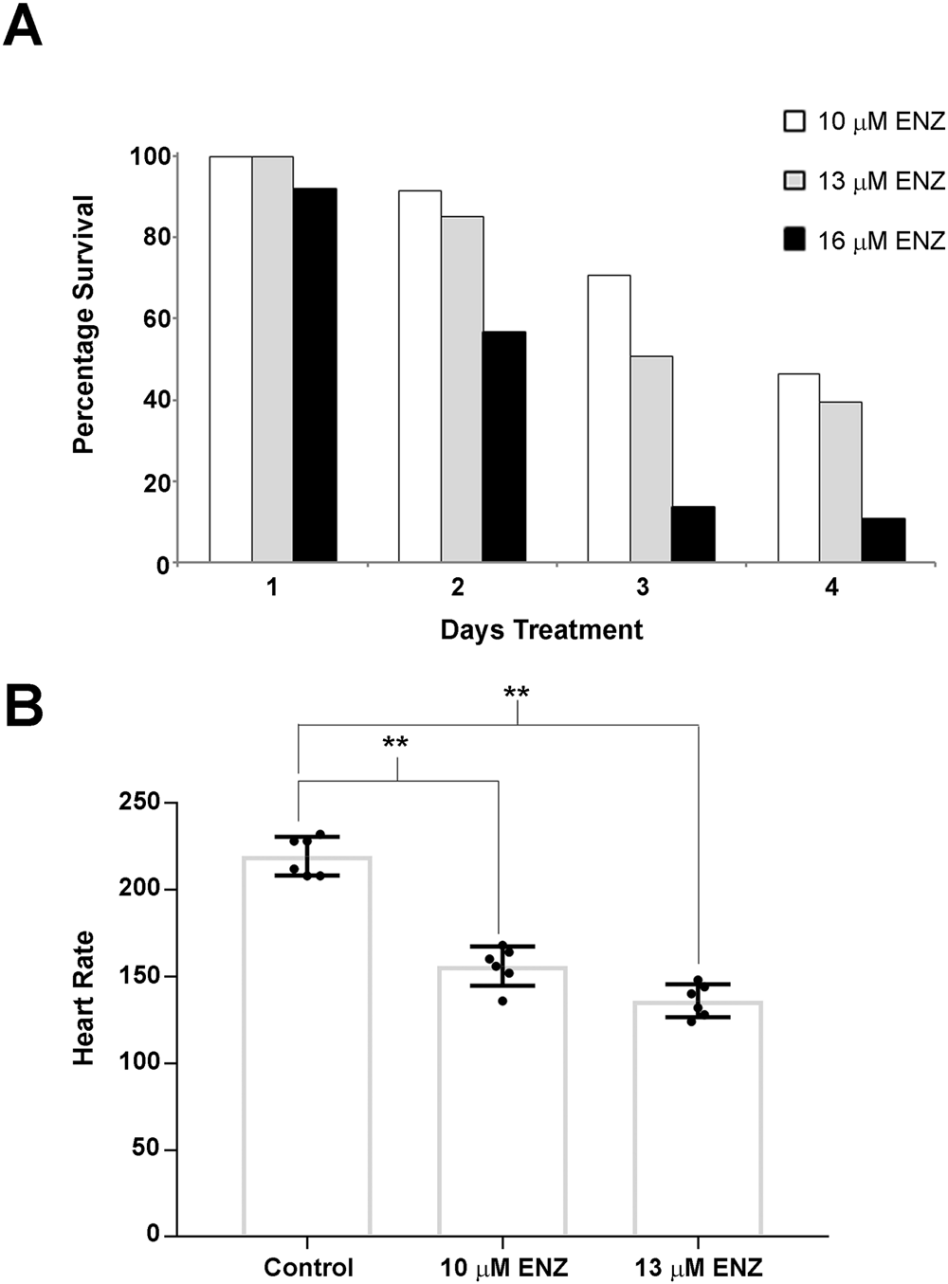
Enzalutamide is toxic to *casper* zebrafish embryos at high doses by inducing bradycardia. (**A**) Percentage survival at 72 hpf of *casper* embryos treated with 10 µM (N = 81 embryos), 13 µM (N = 82 embryos) or 16 µM (N = 74 embryos) of enzalutamide (ENZ) for a total of 4 days. (B) Enzalutamide significantly decreases embryo heart rate (beats per minute – BPM) at 10 µM and 13 µM compared to vehicle control treated embryos, in groups of 30–50 embryos per replicate. Error Bars = Mean ± SEM (N = 6); *P < 0.05, **P < 0.01 for significant differences in heart rate between groups of fish were determined using the Student’s t-test.

**Figure 5:**
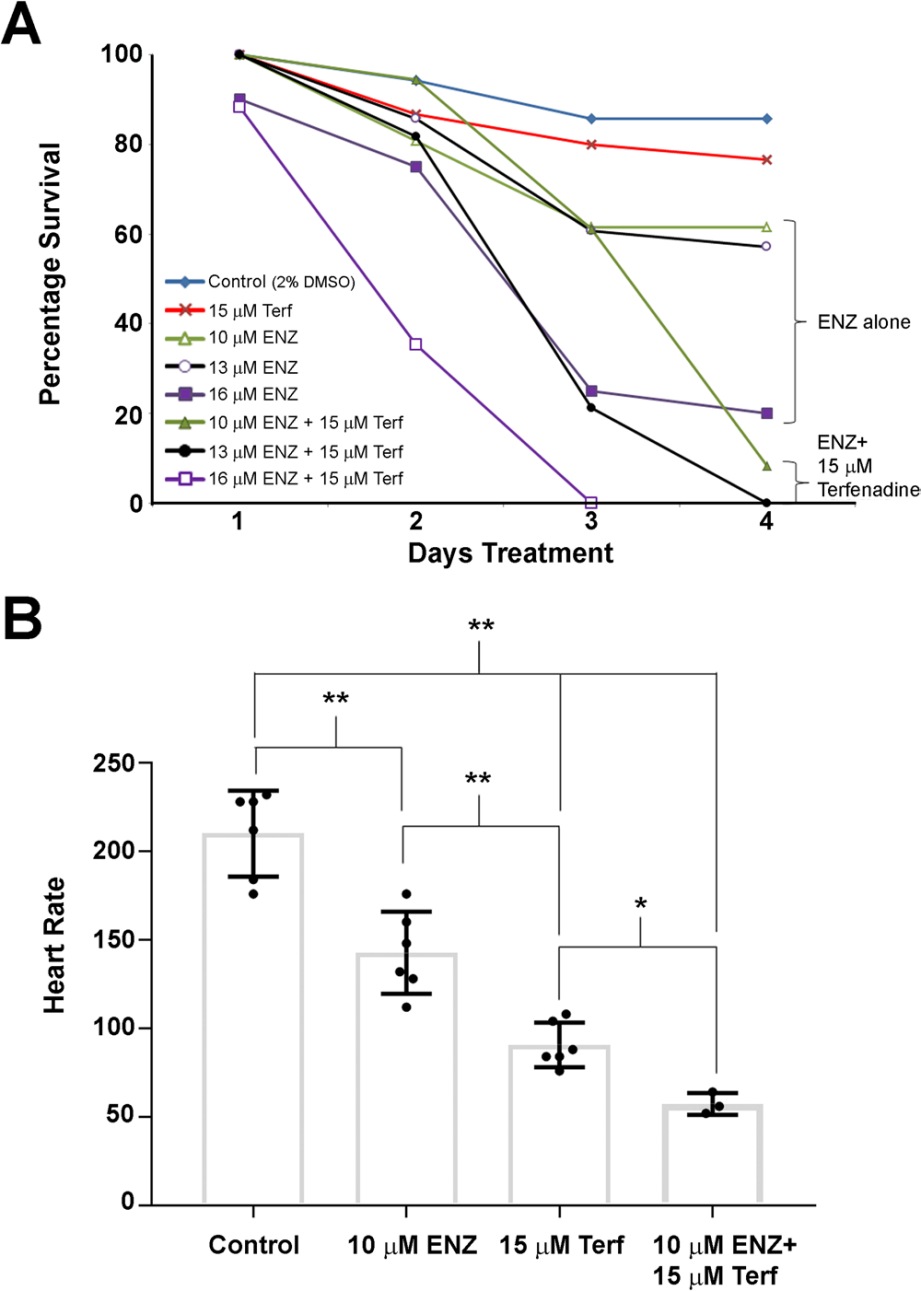
The combination of enzalutamide and terfenadine further decrease heart rate and embryo survival. (**A**) Survival curve of 72 hpf *casper* embryos treated with vehicle (Control, 2% DMSO) or the indicated concentrations of enzalutamide (ENZ) alone, terfenadine (Terf) alone, and both drugs in combination. Compounds were added to the water of groups of 30–50 embryos and percentage survival over time in days is indicated. (**B**) Embryonic heart rates decreased significantly when treated with the combination of enzalutamide and terfenadine. Groups of 30–50 embryos were treated with the indicated concentrations of enzalutamide, terfenadine or a combination of both, and the heart rate was determined on the surviving fish after 4 days. Error Bars = Mean ± SEM (N = 6, with the exception of the enzalutamide + tefenadine group for which only 3 animals survived (N = 3)); *P < 0.05, **P < 0.01 for significant differences in heart rate between groups of fish were determined using the Student’s t-test.

In determining the 72 hour MTD50 for enzalutamide, we observed that administration of higher doses of drug were embryonic lethal to zebrafish. To further study this phenomenon, we treated embryos with 10, 13 or 16 µM enzalutamide for 4 days. We saw a steady decrease in embryo survival over time and relative to dose, peaking at 46% at 10 µM, 40% at 13 µM, and 11% at 16 µM enzalutamide after 4 days of treatment (Fig. 4A). We also monitored heart rate in enzalutamide-treated embryos by counting the number of beats per minute (bpm) of each embryo averaged over 20–30 embryos. We found that embryonic heart rate significantly decreased from 219 ± 5 bpm in vehicle control treated embryos to 156 ± 5 bpm and 136 ± 4 bpm in 24h-treated 10 and 13 µM enzalutamide-treated embryos, respectively (Fig. 4B; p < 0.001). The bradycardia we observed was found in all treated embryos and appeared to be a highly reproducible phenotype that is reminiscent of the cardiac effects of agents that target the potassium voltage-gated channels in zebrafish^18^. Furthermore, only 24 h of drug treatment with 13 µM enzalutamide was sufficient to produce bradycardia as compared to vehicle alone (see Supplemental Movies 1 and 2). Therefore, we sought to determine if we could exacerbate this enzalutamide-induced bradycardia by treating embryos with terfenadine, a well characterized potassium-channel blocking agent^19^. We found that 15 µM terfenadine was sufficient to alter cardiac rhythm within 24 h in 72 hpf embryos (Supplemental Movie 3). We then treated groups of 25–35 embryos at 72 hpf with enzalutamide alone, terfenadine alone (15 µM) or a combination of both enzalutamide and terfenadine with differing concentrations of enzalutamide for a total of 4 days (Fig. 5A). Survival was the lowest in the embryonic group treated with the combination of enzalutamide and terfenadine. For example, all embryos were dead after 3 days of treatment with the combination of 16 µM of enzalutamide and 15 µM of terfenadine, and all embryos were dead after 4 days of treatment with the combination of 13 µM of enzalutamide and 15 µM of terfenadine. To determine if the increased lethality of combined enzalutamide and terfenadine was related to exacerbation of bradycardia, we again examined the heart rate in singly and co-treated animals. We found that the heart rate was significantly decreased in all treated embryos compared to the control group (Fig. 5B; p < 0.001). Terfenadine (15 µM) or enzalutamide (10 µM) alone decreased the heart rate to 91 ± 5 and 143 ± 10 bpm (respectively). However, embryos treated with both enzalutamide and terfenadine exhibited a further decrease in heart rate to only 57 ± 4 bpm, which was significantly different to control (p < 0.001) or singly treated animals (p < 0.03). Thus, enzalutamide and terfenadine can synergize to profoundly inhibit cardiac rhythm in the zebrafish.

## Discussion

Morbidity and mortality associated with prostate cancer remains a significant concern for the aging male population, and appropriate preclinical models for this disease are necessary intermediaries for the evaluation of therapeutic approaches to improve longevity and quality of life in these patients. Currently, men with advanced prostate cancer receive androgen deprivation therapy (ADT) but this treatment is not without side-effects, and intrinsic or acquired resistance to ADT is common^20,21^. ADT has been shown to significantly increase cardiovascular morbidity in prostate cancer patients^22–25^. The use of LHRH agonists increases the rate of cardiac causes of death when compared to the use of LHRH antagonists^26^, but it is unknown if this is due to the LHRH agonist, the use of the androgen blockade, or the combination. Furthermore, it has been shown that all ADTs cause prolongation of the QT interval in Phase III clinical trials^27^. Enzalutamide is used as long-term treatment for men who are refractory to ADT and develop CRPC. Treatment of CRPC with enzalutamide has been shown to prolong life by 4.8 months^28^. Unfortunately, despite treatment, virtually all men diagnosed with CRPC succumb to their disease within two years^29,30^. In the ongoing clinical trial for the safety and efficacy study of enzalutamide in patients with nonmetastatic castration-resistant prostate cancer (PROSPER) in the United States, exclusion criteria include men with clinically significant cardiovascular disease (NCT02003924). As most men with prostate cancer are 65 years or older^31^ and thus likely to have elevated rates of cardiovascular disease, not being able to receive life prolonging treatments like enzalutamide due to cardiovascular complications could significantly impact the management of these patients.

In this report, we demonstrate that the zebrafish XT platform is an ideal *in vivo* model for preclinical screening of ADT and anti-androgen therapies to both limit prostate cancer progression and confirm off-target side effects of these therapies. We have shown that LNCaP cells injected into zebrafish embryos at 48 hpf proliferate significantly over the course of four days with or without testosterone; however, proliferation is significantly increased in embryos treated with 125 nM testosterone (Fig. 1A–C). These studies are in agreement with previous studies indicating that LNCaP cancer cells can propagate in this model^12–14^. However, while these studies concluded that androgen-dependent LNCaP cells could grow and were poorly metastatic in the zebrafish (a phenotype seen in mouse subcutaneous xenografts^32^), these studies were done without supplementation with exogenous testosterone. Our data indicates that the results of these previous studies performed in the absence of testosterone should be interpreted with caution, and all future PCa zebrafish XT experiments should include testosterone to more faithfully recapitulate the hormonal milieu found in patients. Although testosterone did not increase metastatic behavior of either LNCaP or C4-2 cells (data not shown), testosterone did significantly induce LNCaP proliferation *in vivo* and this proliferation could be suppressed in xenotransplanted zebrafish embryos co-treated with 5 µM enzalutamide (Fig. 2). In contrast, exogenous testosterone did not promote the growth of CRPC cell line C4-2 in engrafted embryos nor did enzalutamide inhibit growth, as expected from *in vitro* proliferation studies^17^. These findings demonstrate that enzalutamide is effective at limiting androgen-dependent prostate cancer cell growth but not the C4-2 CRPC cell line in this *in vivo* model, and highlights the opportunities for studying drugs that act on androgen signaling in PCa using the zebrafish.

It should also be noted that treatment with enzalutamide alone, however, did not significantly alter baseline proliferation of LNCaP cells in the absence of exogenous testosterone added to the embryo water. One interpretation of this data, is that levels of testosterone in the embryo itself during our experimental time frame of 48–144 hpf are likely insufficient to trigger AR-dependent growth. Nonetheless, levels of testosterone can be easily manipulated in the zebrafish XT model by adding different concentrations of androgen to the aquatic medium, and the short term nature of experiments in zebrafish compared to mice (days versus weeks) removes any confounding changes in endogenous testosterone that might occur during development and obviates the need for physical castration as would be required for mouse xenograft experiments.

In previous PCa xenograft studies using the zebrafish, both androgen-dependent LNCaP and androgen-independent cell lines such as PC3 and DU145 cell lines have been used to study PCa growth in fish in the absence of exogenous testosterone^12–14,33^. Although it is difficult to predict the behavior of the PC3 and DU145 cell lines in response to exogenous testosterone in the zebrafish xenograft model *a priori*, we note that models of CRPC that over-express the AR, such as the LNCaP-AR cell line that is responsive to enzalutamide when engrafted in mice^34^, would likely respond to enzalutamide treatment after engraftment in the zebrafish due to their reliance on the AR for their growth. Taken together, these data indicate that zebrafish XT represents an attractive platform in which to examine androgen signaling in PCa cells and the effects of anti-androgen treatments in arguably a more cost-efficient model than mice.

We also observed in our studies that treatment of zebrafish embryos with increasing amounts of enzalutamide caused a dose-dependent pattern of mortality from 10 to 16 µM over a 4 day treatment period (Fig. 4A). While 10 µM enzalutamide did not cause pronounced mortality over this treatment period, significant bradycardia was observed in this group as well as the 13 µM treated embryos (Fig. 4B). The heart rates of surviving embryos from the 16 µM fish were not included in this analysis due to significant overall toxic effects including cardiac edema that likely would exacerbate the cardiac phenotype (data not shown). These cardiac outcomes are interesting in that they reflect a direct effect of this anti-androgen compound on heart rate, in the absence of an LHRH agonist, which has not been previously examined in human patients. Sex hormones have well-documented effects on the cardiovascular system, including the control of proper contractile rhythms of the heart^35,36^. A pathologically long QT interval, or Long QT Syndrome (LQTS), is associated with improper cardiac pacing, arrhythmias, and sudden cardiac death. Interestingly, all individuals are born with a similar QT interval, but it begins shortening in males during puberty and is about 6% shorter than the female QT interval until it begins lengthening again later in life to match the female QT interval by 50 years of age^36^. Testosterone has indeed been linked to this change in QT length; men with hypogonadism^37^ and decreases in serum testosterone due to advanced age^38^ are more likely to have prolonged QT intervals. This could be due to changes (including transcriptional) in ion channels necessary for the proper repolarization of the ECG, including potassium and calcium channels^39^. While we are not able to directly measure the QT interval of embryonic zebrafish, the addition of terfenadine to the enzalutamide treated fish caused increased mortality and further decreased embryo heart rate when combined with enzalutamide (Fig. 5A,B). Terfenadine is an antihistamine drug which is a potent inducer of acquired long QT syndrome by blocking the primary delayed rectifier current (I_Kr_) in the human ventricle, hERG^19^. Our results suggest that blocking this channel while concurrently treating with enzalutamide exacerbates the toxic and cardiac effects of this anti-androgen. The potential for adverse cardiac events has already been recognized by the developers of enzalutamide, as pre-existing heart conditions including long QT syndrome remain a primary exclusion criteria for clinical trials of this drug^31^. However, to our knowledge, our study is the first to describe direct cardiac effects of enzalutamide in vertebrates. Altogether, our results support existing evidence that significant cardiac rhythm disturbances are possible with enzalutamide treatment, and patients should be closely monitored for this toxicity regardless of pre-existing conditions.

When considering the relevance of any human tumour xenograft model, it is important to appreciate that often there is discordance between effective drug doses in the animal models and actual human patients. This has been shown to be particularly the case in mouse and dog models^40^. Despite this caveat, the dose of enzalutamide used in this study approached the range of serum levels found in human patients. For example, typical patient serum levels of enzalutamide range from 12–20 μg/ml (or 26–43 μM)^41^. We treated engrafted zebrafish embryos with 5 μM enzalutamide. However, we could not treat animals with more than 16 μM drug without significant morbidity. Whereas for testosterone, we treated engrafted embryos at the MTD50 of 125 nM hormone; a dose which is more than 45 times the typical human prostate tissue concentration of ~2.7 nM^42^.

Finally, mitigating the off target toxic effects of chemotherapies while maintaining the efficacy of the cancer treatment is an increasingly important area of research as cancer survivorship increases; and in the case of PCa, long-term therapies such as ADT can extend life for a decade or more. Importantly, the zebrafish has been validated as a model for the discovery of protective agents against chemotherapy-induced cardiotoxicity. For example, cardiac effects of the common anthracycline chemotherapeutic, doxorubicin, have been recapitulated in the embryonic zebrafish model. Using this model, two compounds, visnagin and diphenylurea, were able to rescue bradycardia and reduced circulation phenotypes in zebrafish treated with doxorubicin^15^. This has important implications in the current context of ADT and prostate cancer, the goal of which would be to suppress tumour progression while preventing significant and potentially life threatening cardiac events. With respect to PCa research we believe the zebrafish XT model holds great promise for similar screens, whereby simultaneous evaluation of PCa cytotoxicity and cardiotoxicity can be conducted to reveal novel anti-androgens that do not impact cardiac function, or alternatively “protective” compounds that enable the safer delivery of enzalutamide.

## Methods

### Zebrafish Husbandry

Adult *casper* ^43^ and Tg(*myl7*::*eGFP*)/*casper* (generated by crossing Tg(*myl7::eGFP*) fish – a gift from Dr. Ian Scott, SickKids, Toronto, Canada with *casper* fish) zebrafish were maintained in a recirculating commercial housing system (Pentair, Apopka, FL) at 28 °C in 14 h:10 h light:dark conditions. *casper* and Tg(*myl7::GFP*)/*casper* zebrafish were bred according to standard protocol^44^, and embryos were collected and grown in E3 medium (5 nM NaCl, 0.17 mM KCl, 0.33 mM CaCl_2_, 0.33 mM MgSO_4_) in 10 cm Petri dishes until the desired time point. Embryos were cleaned and provided with new media every 24 hrs. Use of zebrafish in this study was approved by and carried out according to the policies of the Dalhousie University Committee on Laboratory Animals (Protocol #15–126 and #15–134).

### Cell culture and Xenotransplantation

LNCaP prostate cancer cells were purchased from ATCC, and the androgen-independent C4-2 subline of LNCaP cells were kindly provided by Dr. Leland W.K. Chung (1992, MDACC, Houston Tx) and tested and authenticated by whole-genome and whole-transcriptome sequencing on Illumina Genome Analyzer IIx platform in July 2009. LNCaP cells were cultured in RPMI-1640 and C4-2 cells were grown in DMEM medium, both were supplemented with 10% fetal bovine serum (FBS) and 1% penicillin/streptomycin (Gibco). Prior to xenotransplantation, five million LNCaP (or C4-2) cells were grown to 80% confluence and trypsinized with 0.25% trypsin (Gibco) for 3–5 minutes. Cells were then washed with culture medium and centrifuged for 5 minutes at 300 *g*. Cells were resuspended in 10 mL of phosphate-buffered saline (Gibco) (PBS) and with 5 µg/mL Cell Tracker Orange CMTMR Dye (ThermoFisher)^15^. The cell suspension was incubated for 20 minutes at 37 °C. Cells were pelleted, washed twice with culture medium and once with PBS, and resuspended in 100 µL culture medium.

Naturally dechorinated 48 hours post-fertilization (hpf) *casper* zebrafish embryos were anesthetized with 0.09 mg/mL tricaine (Sigma-Aldrich) and used for cell transplantation using protocols described previously^10,45^. The fluorescently labeled cells were loaded into a pulled capillary needle for embryo injection. A PLI-100A Pico-injector microinjection system (Harvard Apparatus, Holliston, MA) was used to manually inject 50–100 cells into the yolk sac of each embryo. Following injections, embryos were kept at 28 °C for 30 minutes and at 35 °C for the duration of the experiments.

### Zebrafish MTD50 Assays

48 hpf zebrafish embryos were treated with increasing concentrations of each drug for 72 hours to ascertain toxicity, and treatment dose for further experiments was chosen based on half the maximum tolerated dose (MTD50) of the embryos during 72 hour treatment^10^.

### *Ex vivo* Tumour Cell Quantification

Groups of 30–40 xenografted embryos were treated with 125 nm testosterone (Sigma-Aldrich) alone, 5 µM enzalutamide (MDV3100) (Selleckchem), in combination with 125 nm testosterone and 5 µM enzalutamide, or vehicle (1% DMSO) by addition to the water. At 24 hours post-injection and 72 hours post-treatment, 20 embryos from each group were euthanized by a tricaine overdose (1 mg/mL). For each replicate (N = 4), embryos were dissociated in 100 mg/mL collagenase (Sigma-Aldrich) solution for 30 minutes. Upon completion, 200 µL of 100% FBS was added to slow the enzymatic reactions occurring between the dissociation mix and the embryo cell suspension. The suspension was then centrifuged for 5 minutes at 300 *g* and the supernatant removed, leaving the pellet of fluorescent human tumour cells among the zebrafish cells. The cells were finally resuspended in 10 µL per embryo of PBS-FBS (10% FBS) solution for imaging. Ten 10 µL boli were pipetted onto a glass slide as a hanging droplet and imaged under a DsRed filter as a 2 × 3 mosaic under a 5x objective^10^. The dissociated samples were imaged using an inverted Axio Observer Z1 microscope (Carl Zeiss, Westlar, Germany) and images were analyzed using ImageJ software (NIH, Bethesda, MD, USA) where relative fluorescent cell numbers were determined per group.

### Enzalutamide and Terfenadine Toxicities and Heart Rate Analysis

Embryos from pooled pairwise Tg(*myl7*::*eGFP*)/*casper* fish crosses were reared at 28 °C in Petri dishes containing E3 medium, as described above, until 72 hpf. Working stocks of 25 mM terfenadine (Sigma-Aldrich) and 100 mM enzalutamide (Selleckchem) were prepared in 100% DMSO and stored in individual aliquots at −20 °C until use. All drug treatments were prepared to their final concentration in E3 medium supplemented with DMSO to a final concentration of 1%. Groups of 20–50 fish were placed in 10 cm Petri dishes with 25 mL of the treatment solution (enzalutamide, terfenadine, or both) in the indicated concentrations (10, 13, or 16 µM enzalutamide and/or 15 µM terfenadine, as well as 1% DMSO vehicle controls). Fish were treated for four days, from 72 to 168 hpf, at 35 °C. For toxicity measurements, plates were checked every 24 hours and dead embryos were counted and removed from the dishes. Cumulative mortality was then calculated over the four day treatment period. Heart rates were measured in embryos randomly selected from each dish on the last day of treatment (168 hpf). Anesthetic water (0.2 mg/mL tricaine) was prepared in E3 egg medium and heated to 35 °C in the embryo incubator. Embryos were removed one at a time from the incubator and placed in a small volume of the anesthetic egg water for approximately 30 sec prior to the heart rate measurement. Hearts were visualized with green fluorescence using a stereo microscope (Zeiss Discovery.v20), and the beats of the ventricle were counted over a 15 sec interval. These values were multiplied by 4 to achieve the final value of beats per minute (bpm). Results from a minimum of 3 independent experiments were aggregated and mortality was represented as a percentage of total embryos treated. For video recordings, serial images of representative control vehicle treated embryos and embryos treated with 13 µM enzalutamide, or 15 µM terfenadine for 24 h were taken every 200 ms on an inverted compound microscope (Zeiss Axio Observer Z1) equipped with a Zeiss AxioCam HR R3. Images were then converted to 5 frames/s.avi files using Zen software (Zeiss) and edited using Pinnacle Studio 19 to produce movies time scaled to 25 frames/s running at ~0.3 X actual speed.

### Statistical Analysis

All data was tabulated and analyzed using Excel 2010 (Microsoft) and figures generated with Graphpad Prism 7.01. All experiments were performed at least in triplicate (as indicated in figure legends) and the Student’s T-test was used to determine statistical significance between groups.

## Electronic supplementary material

Supplementary Information

Movie S1

Movie S2

Movie S3
