# Supplementary material for: Enzalutamide inhibits testosterone-induced growth of human prostate cancer xenografts in zebrafish and can induce bradycardia
Source: Sci Rep. 2017 Oct 31;7:14698. doi: 10.1038/s41598-017-14413-w (PMC5665934; doi:10.1038/s41598-017-14413-w)
Supplement: Supplementary file 1 — Supplementary Information [file 41598_2017_14413_MOESM1_ESM.pdf]

Enzalutamide inhibits testosterone-induced growth of human prostate cancer xenografts in zebrafish and can induce bradycardia

Nicole Melong[1,2], Shelby Steele[3], Morgan MacDonald[4], Alice Holly[2], Colin C. Collins[5], Amina Zoubeidi[5], Jason N. Berman[1,2,3*], and Graham Dellaire[2*]

1. Department of Pediatrics, IWK Health Centre
2. Department of Pathology, Dalhousie University
3. Department of Pediatrics, Dalhousie University
4. Undergraduate Program, Faculty of Medicine, Dalhousie University
5. Vancouver Prostate Centre, Vancouver British Columbia

*Correspondence: G. Dellaire (dellaire@dal.ca) and J.N. Berman (Jason.Berman@iwk.nshealth.ca)

Supplementary Information

Movie S1. Heart rate in a control Tg(*myl7*::*eGFP*)/*casper* zebrafish embryo.
A control 72 h post-fertilization Tg(*myl7*::*eGFP*)/*casper* zebrafish embryo is shown after 24 h treatment with vehicle (1% DMSO). The movie is 25 frames/s and runs at 0.3 X actual speed.

Movie S2. Heart rate in a Tg(*myl7*::*eGFP*)/*casper* zebrafish embryo treated with 13 μM enzalutamide. A 72 h post-fertilization Tg(*myl7*::*eGFP*)/*casper* zebrafish embryo is shown after 24 h treatment with 13 μM enzalutamide. The movie is 25 frames/s and runs at 0.3 X actual speed.

Movie S3. Heart rate in a Tg(*myl7*::*eGFP*)/*casper* zebrafish embryo treated with 15 μM terfenadine. A 72 h post-fertilization Tg(*myl7*::*eGFP*)/*casper* zebrafish embryo is shown after 24 h treatment with 15 μM terfenadine. The movie is 25 frames/s and runs at 0.3 X actual speed.
